# Supplementary material for: Exopolysaccharide Carbohydrate Structure and Biofilm Formation by Rhizobium leguminosarum bv. trifolii Strains Inhabiting Nodules of Trifolium repens Growing on an Old Zn–Pb–Cd-Polluted Waste Heap Area
Source: Int J Mol Sci. 2021 Mar 10;22(6):2808. doi: 10.3390/ijms22062808 (PMC7998805; doi:10.3390/ijms22062808)
Supplement: Supplementary file 1 [file ijms-22-02808-s001.zip › ijms-1121998-supplementary.pdf]

Figure S1. Mass spectra of peaks of not identified carbohydrate-type compounds detected by GC-MS analysis of exopolysaccharides of *R. leguminosarum* bv. *trifolii* isolated from root nodules of *T. repens* growing on 100-yrs old Zn-Pb waste heap Bolesław (WH) and non-polluted reference grassland Bolestraszyce (R) not exposed (control) and exposed to 0.05 mM and 1.0 mM Cd, 0.5 mM and 2.5 mM Zn and 0.5 mM and 2.5 mM Pb. Mass spectra of peaks with particular retention indices (*I*) are marked with letters (A-H) as follows: A, mass spectrum of a peak with *I*=1883; B, mass spectrum of a peak with *I*=1896; C, mass spectrum of a peak with *I*=1912; D, mass spectrum of a peak with *I*=1930; E, mass spectrum of a peak with *I*=1937; F, mass spectrum of a peak with *I*=1952; G, mass spectrum of a peak with *I*=1955; H, mass spectrum of a peak with *I*=1958.

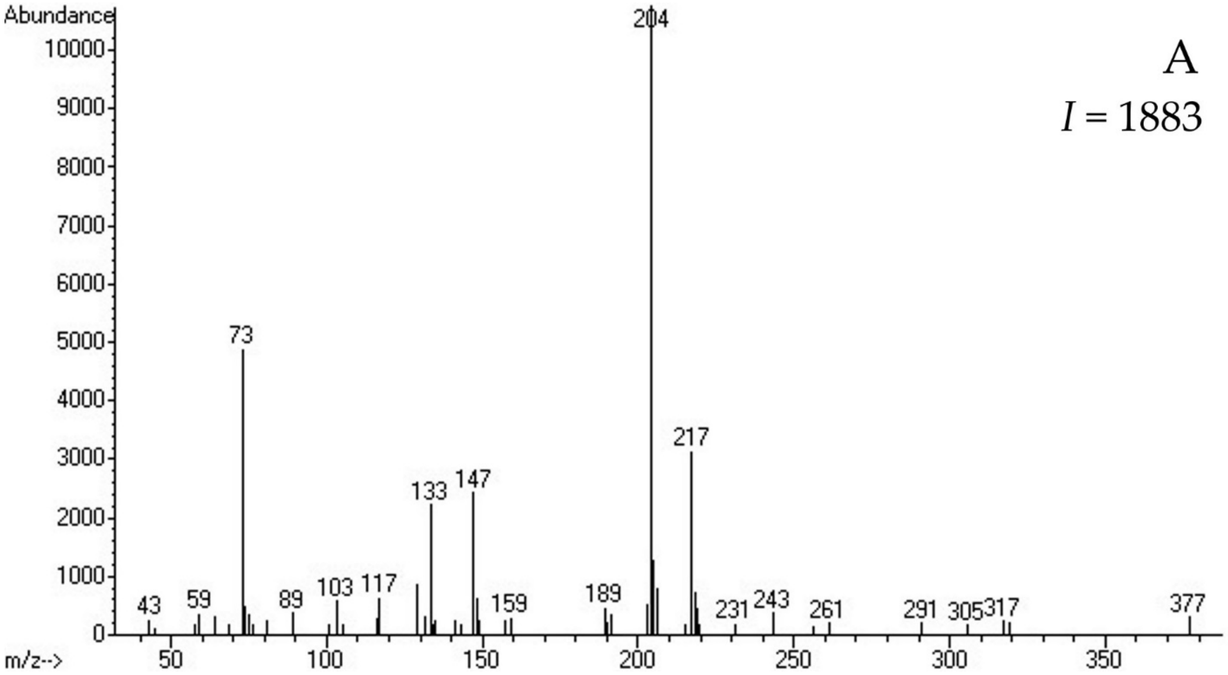

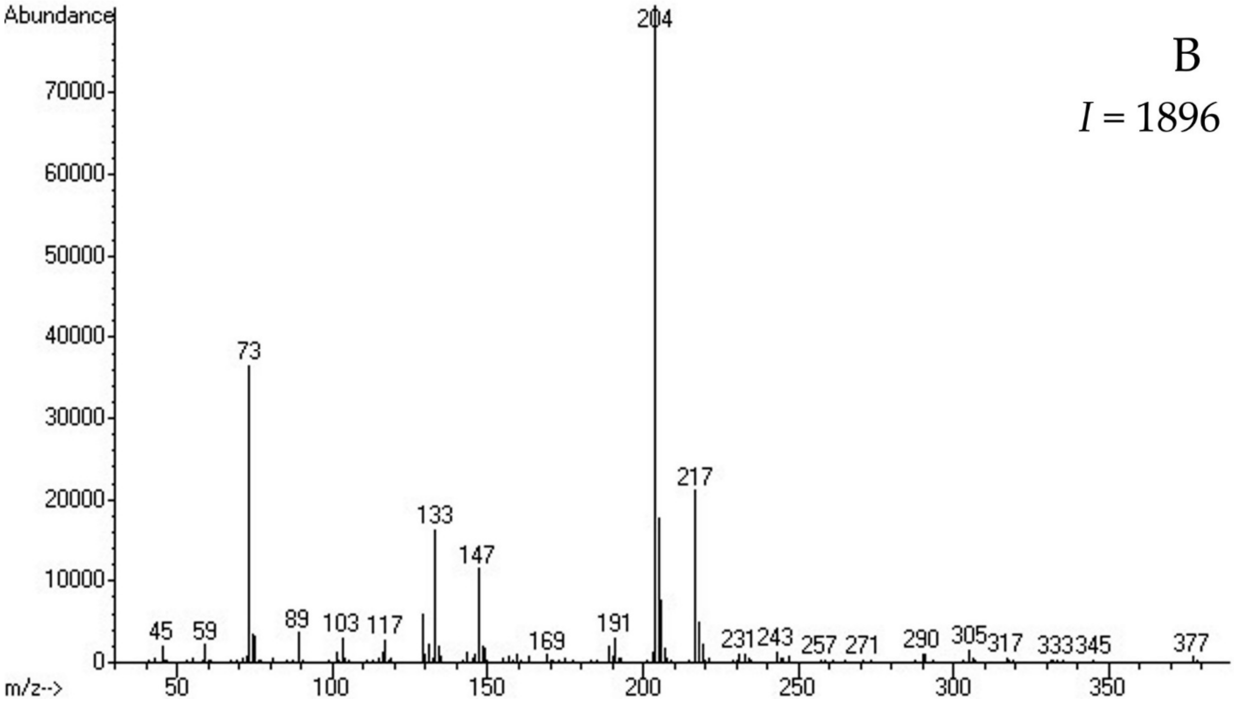

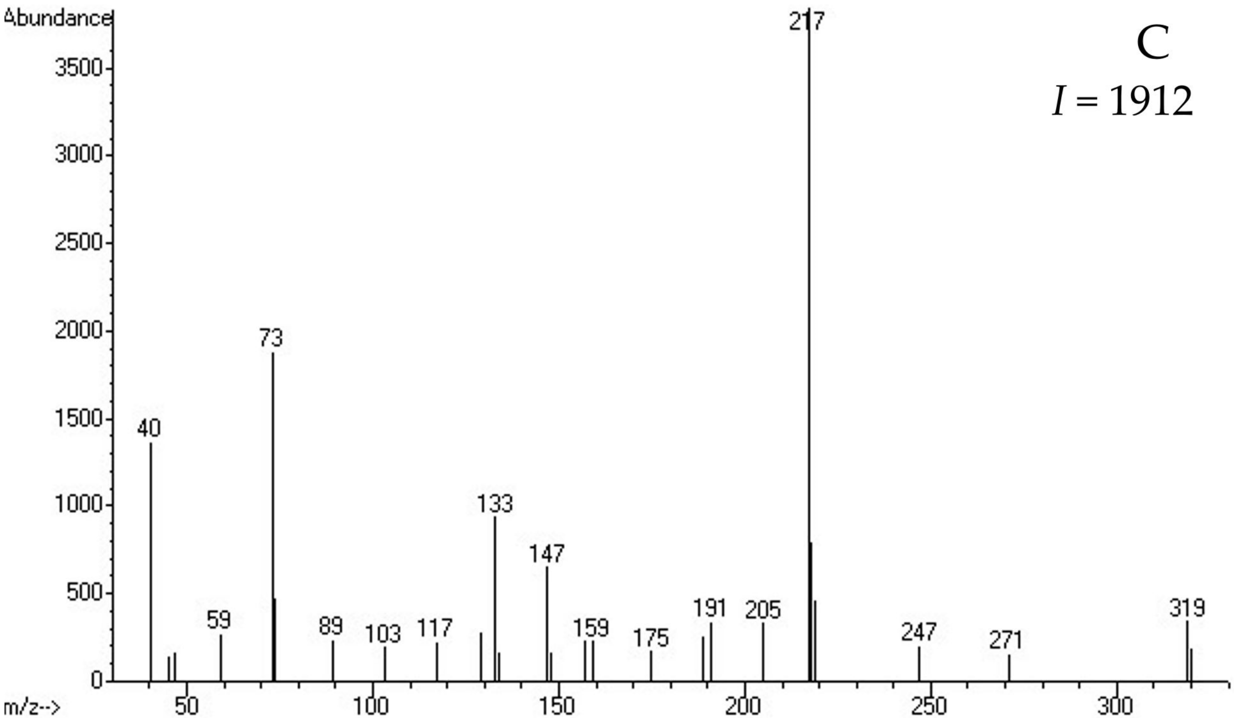
Abundance
3500
3000
2500
2000
1500
1000
500
0
m/z-->
50
100
150
200
250
300
217
73
40
59
89
103
117
133
147
159
175
191
205
247
271
319
C
I = 1912

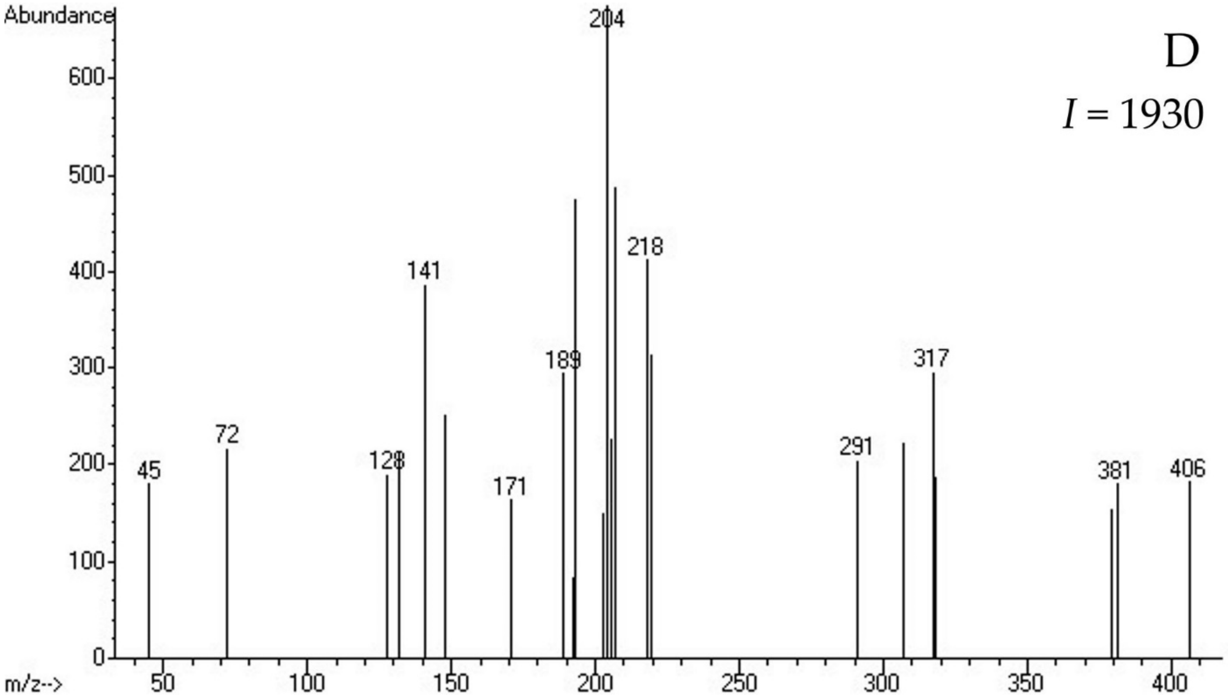
Abundance
600
500
400
300
200
100
0
m/z-->
50
100
150
200
250
300
350
400
204
218
141
189
317
45
72
128
171
291
381
406
D
I = 1930

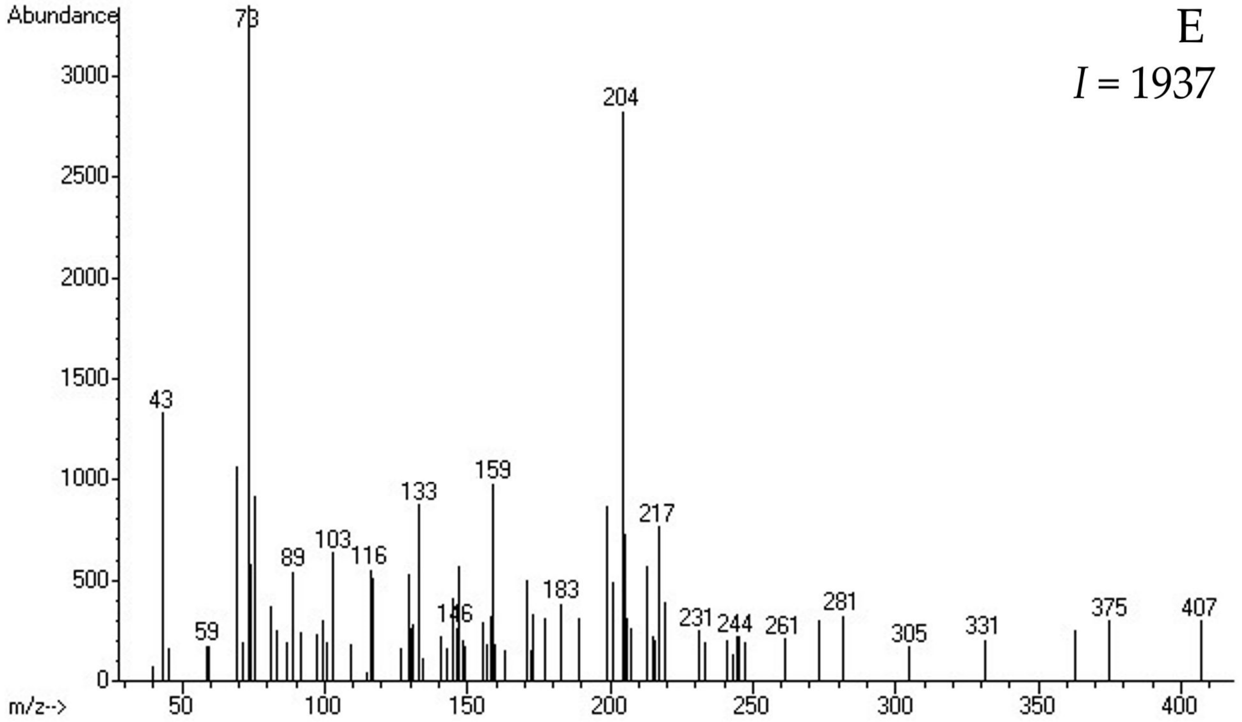
E
I = 1937
Abundance
m/z-->
73
204
43
59
89
103
116
133
146
159
183
217
231
244
261
281
305
331
375
407

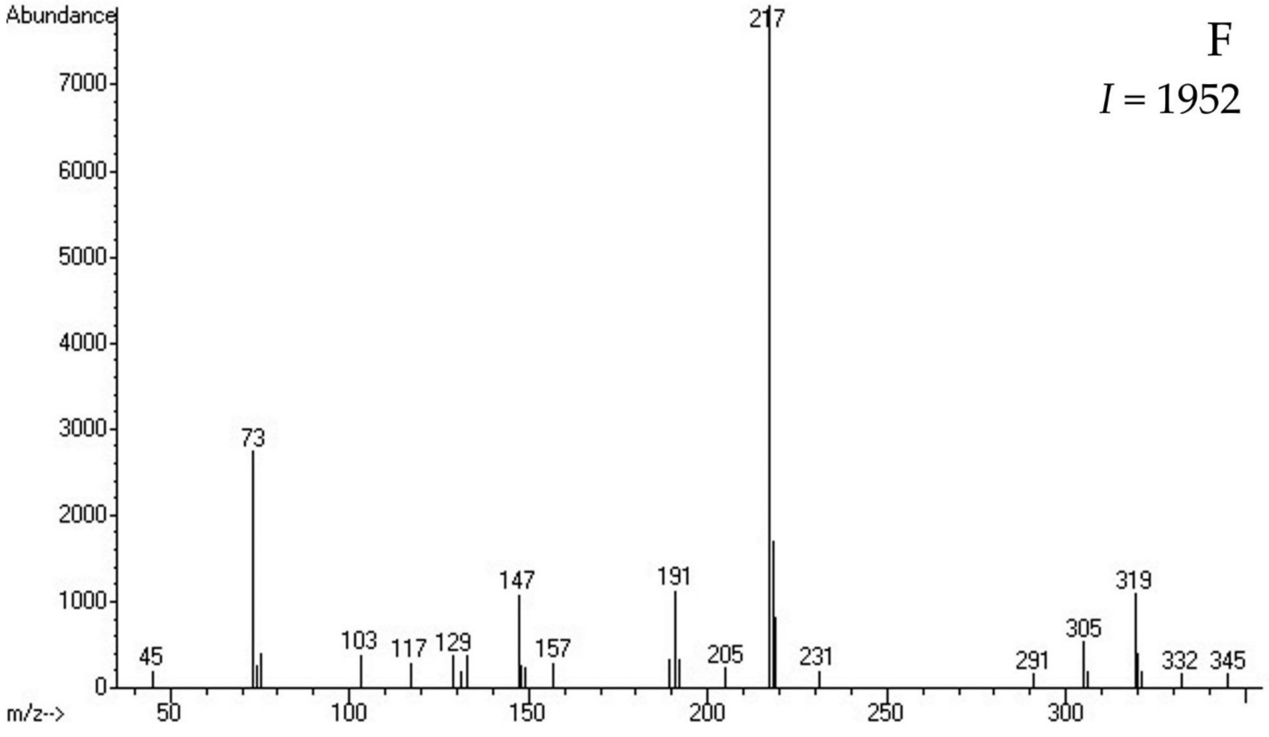
F
I = 1952
Abundance
m/z-->
217
73
45
103
117
129
147
157
191
205
231
291
305
319
332
345

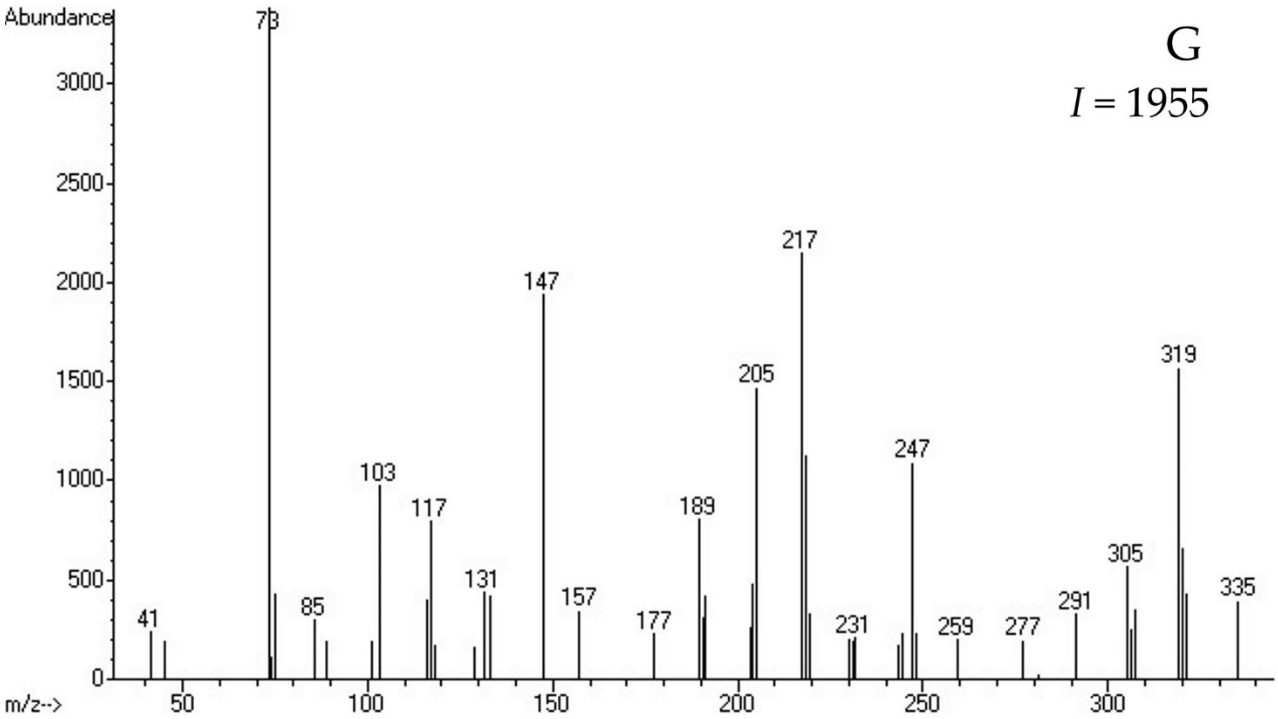
G
I = 1955
Abundance
m/z-->
73
41
85
103
117
131
147
157
177
189
205
217
231
247
259
277
291
305
319
335

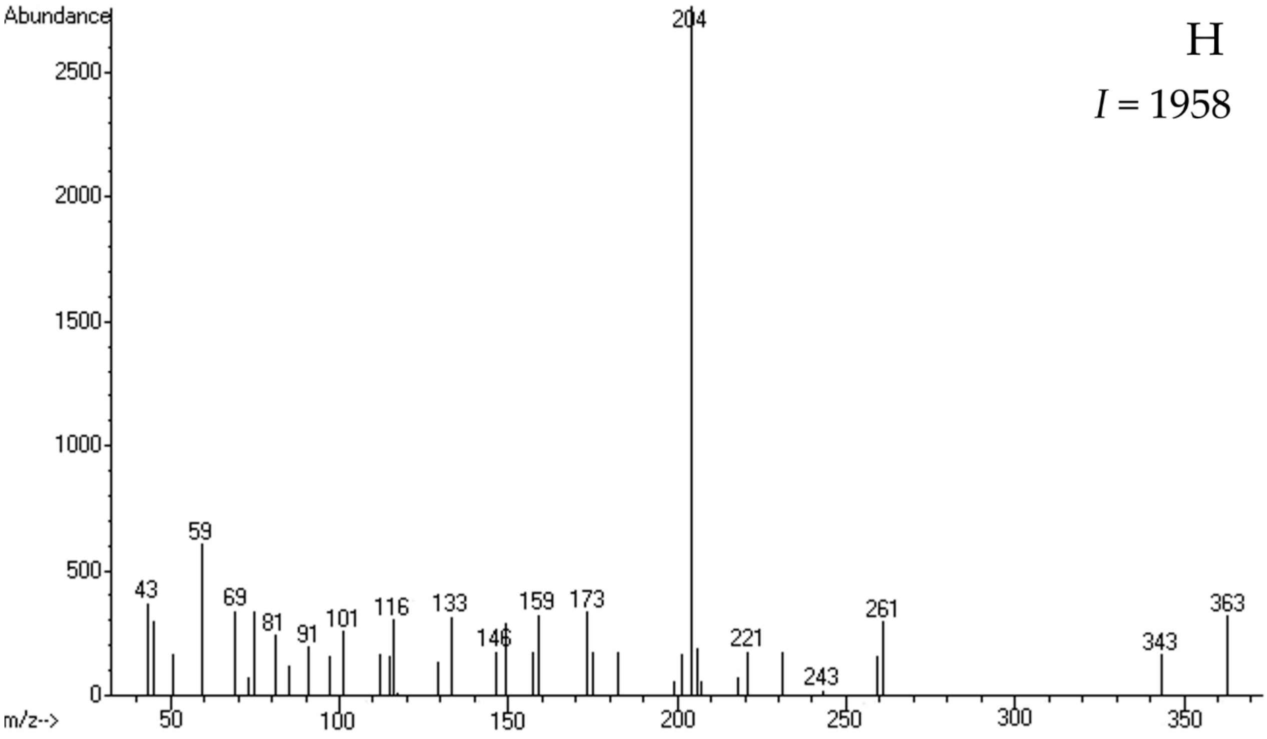
H
I = 1958
Abundance
m/z-->
204
43
59
69
81
91
101
116
133
146
159
173
221
243
261
343
363

Table S1. Mean retention times (t_{Rmean}), determined experimentally (*I*) and reference (I_{REF}) indices of retention, and the mass to charge ratio (*m*/*z*) of compounds detected in exopolysaccharides of *R. leguminosarum* bv. *trifolii* isolated from root nodules of *T. repens* growing on 100-yrs old Zn-Pb waste heap Bolesław (WH) and non-polluted reference grassland Bolestraszyce (R) not exposed (control) and exposed to 0.05 mM and 1.0 mM Cd, 0.5 mM and 2.5 mM Zn and 0.5 mM and 2.5 mM Pb.

Compound	t_{Rmean}	*I*	I_{REF}	*m*/*z*
α-D,L-lyxopyranose	19.175	1597	1603	217,73,204
α-D-arabinopyranose	19.986	1609	1610	204,73,133
D-rhamnose	23.328	1656	1657	204,73,40
L-rhamnose monohydrate	23.683	1661	standard	204,73,191
levoglucosan	27.724	1716	1724	73,217,204
6-deoxy-D-glucose	35.031	1807	1808	204,73,217
methyl α-D-mannopyranoside	36.108	1820	1822	204,73,217
galactofuranoside	37.221	1833	1830	217,73,147
α-D-mannopyranose	38.252	1846	1844	204,73,217
D-fructose	38.685	1851	standard	204,73,217
β-D-galactopyranoside	39.899	1865	1865	204,73,217
α-D-galactofuranose	40.181	1869	1867	217,73,319
α-D-mannofuranose	40.619	1874	1874	217,73,147
α-glucofuranose	41.209	1881	1880	204,217,73
NI (α-talofuranose/β-fructopyranose)	41.414	1883	1882/1887	73,217,147
NI (α-D-fructopyranose/α-D-galactopyranose/β-	42.514	1896	1902/1899/1900	217,73,147
D-galactose	43.273	1905	standard	204,73,217
NI (γ-glucuronolactone/α-D-fructopyranose)	43.847	1912	1902/1913	217,73,147
α-D-glucopyranoside	44.492	1919	1922	204,73,191
α-D-glucopyranose	45.135	1927	1930	204,73,217
NI (δ-tagatose/galacturonic acid)	45.419	1930	1934/1936	204,191,148
D-glucose	45.742	1934	standard	243,73,204
NI (δ-tagatose/galacturonic acid)	46.006	1937	1934/1936	204,133,217
β-D-galactopyranose	46.282	1940	1941	204,73,217
β-D-mannopyranose	46.560	1943	1942	204,217,73
β-talopyranose	46.949	1948	1943	243,73,204
NI (β-D-glucopyranuronic acid/ gulonic acid/	47.280	1952	1957/1958/1954	133,204,73
NI (β-D-glucopyranuronic acid/ gulonic acid/	47.532	1955	1957/1958/1954	73,217,147
NI (β-D-glucopyranuronic acid/ gulonic acid/	47.798	1958	1957/1958/1954	204,117,43
sedoheptulose	48.747	1969	1972	319,73,205
glucitol	49.174	1974	1981	319,73,205
β-D-glucopyranose	53.635	2026	2031	204,73,191
galactonic acid	54.798	2040	2039	204,73,191
gluconic acid	55.073	2043	2043	243,40,73
glucuronic acid	56.117	2056	2056	191,73,204
D-glucuronic acid	59.511	2096	standard	73,147,205
myo-inositol	62.003	2126	standard	217,73,204
N-acetyl-D-glucosamine	62.866	2136	2136	173,73,131

Not identified compounds were marked as NI, and in parenthesis a possible quality identification was proposed.

Table S2. Frequencies (*f*), the percent composition (% *C*), and concentration of identified carbohydrates in exopolysaccharides of *R. leguminosarum* bv. *trifolii* isolated from root nodules of *T. repens* growing on 100-yrs old Zn-Pb waste heap Bolesław (WH) and non-polluted reference grassland Bolestraszyce (R) not exposed (control) and exposed to 0.05 mM and 1.0 mM Cd, 0.5 mM and 2.5 mM Zn and 0.5 mM and 2.5 mM Pb.

Carbohydrate	*f* (%)	% *C*	Mean % *C*	Median % *C*	Concentration (μg mL^{-1})	Mean concentration
α-D,L-lyxopyranose	1.22	0.25	0.25	0.25	15.13	15.13
α-D-arabinopyranose	9.76	2.56-0.04	0.55	0.205	101.18-17.78	31.66
D-rhamnose	1.22	0.04	0.04	0.04	6.69	6.69
L-rhamnose monohydrate	2.44	0.13-0.10	0.115	0.115	12.02-6.41	9.22
levoglucosan	10.98	2.58-0.67	1.25	1.19	19.65-17.59	18.55
6-deoxy-D-glucose	7.32	4.82-2.19	3.25	3.115	60.21-43.27	54.36
methyl α-D-mannopyranoside	69.51	3.86-0.07	1.58	1.6	102.31-18.03	46.89
galactofuranoside	13.41	3.70-0.03	0.70	0.31	21.94-12.61	18.61
α-D-mannopyranose	57.32	3.84-0.05	0.28	0.11	23.10-18.06	19.76
D-fructose	63.41	2.00-0.05	0.35	0.16	49.80-11.64	17.94
β-D-galactopyranoside	40.24	46.04-0.05	2.98	0.73	194.15-12.90	45.43
α-D-galactofuranose	2.44	0.09-0.05	0.07	0.07	19.28-18.80	19.04
α-D-mannofuranose	7.32	2.52-0.09	0.58	0.2	18.05-11.70	17.57
α-glucofuranose	10.98	1.14-0.07	0.43	0.45	24.33-17.50	20.11
D-galactose	24.39	15.42-0.05	4.28	0.535	288.64-18.52	75.58
α-D-glucopyranoside	84.15	16.49-0.11	2.26	1.05	365.25-17.49	39.83
α-D-glucopyranose	7.32	4.88-3.75	4.46	4.585	92.40-49.74	70.86
D-glucose	34.15	5.39-0.05	1.39	0.295	124.85-17.71	34.34
β-D-galactopyranose	59.76	5.8-0.06	0.50	0.22	158.63-15.51	28.39
β-D-mannopyranose	18.29	1.00-0.07	0.20	0.11	29.83-17.84	19.79
β-talopyranose	32.93	11.54-0.1	3.70	2.79	297.57-18.69	83.46
sedoheptulose	24.39	10.96-0.04	2.68	0.85	300.52-11.17	82.98
glucitol	98.78	100.00-53.86	90.50	91.08	1816.58-34.37	731.57
β-D-glucopyranose	23.17	10.31-0.36	4.59	5.29	42.22-17.68	24.79
galactonic acid	25.61	5.42-0.09	0.51	0.15	4346.71-42.89	341.89
gluconic acid	12.20	1.77-0.04	0.71	0.62	71.37-45.35	51.90
glucuronic acid	20.73	1.02-0.04	0.31	0.18	155.82-38.68	74.87
D-glucuronic acid	1.22	0.08	0.08	0.08	41.91	41.91
myo-inositol	1.22	0.013	0.13	0.13	26.93	26.93
N-acetyl-D-glucosamine	2.44	0.09-0.08	0.085	0.085	19.86-17.52	18.69

Figure S2. Standard curves of D-(-)fructose, L-rhamnose monohydrate, D-(+)-galactose, D-glucuronic acid, D-(+)-glucose, *myo*-inositol, D-(+)-galacturonic acid monohydrate, D-(+)-xylose monosaccharide solutions prepared as a mixture of particular standard monosaccharides in concentrations as follows 10 µg mL^{-1}, 50 µg mL^{-1}, 100 µg mL^{-1}, 200 µg mL^{-1}, and 500 µg mL^{-1}.

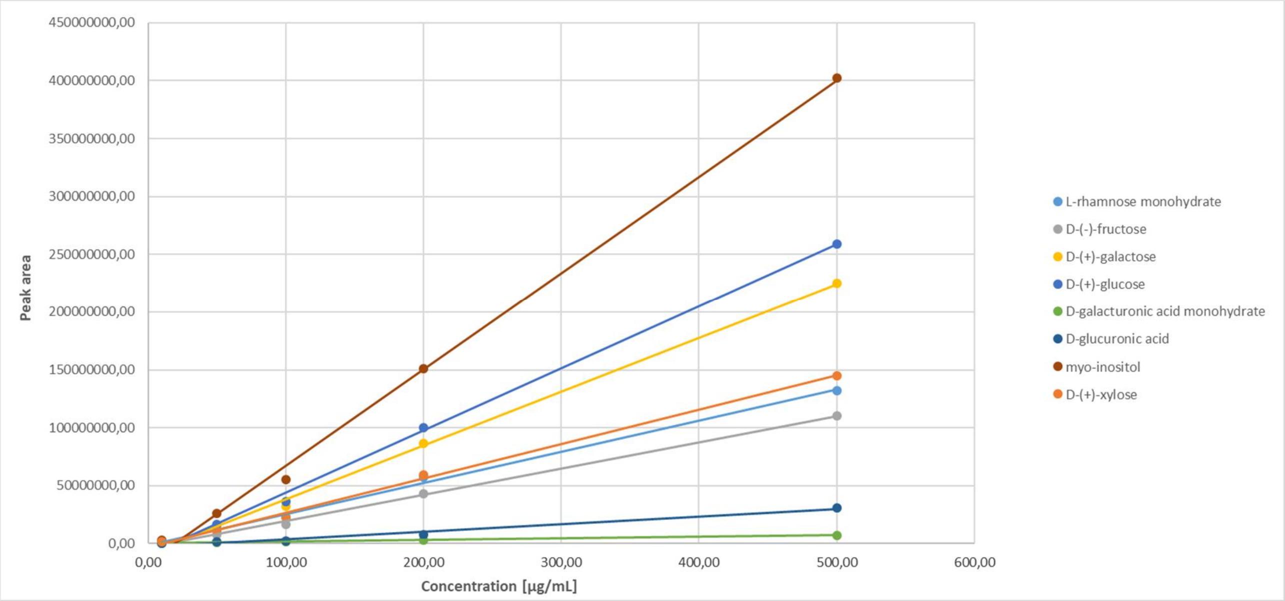

Table S3. Analytical characteristics of the GC-MS method used for quantification of carbohydrates.

**Carbohydrate standard**	Linear range (µg mL^{-1})	Calibration curve equation y=(a±SD)x+(b±SD), n=3	R^2	*CV* (%)	*LOD* (µg mL^{-1})	*LOQ* (µg mL^{-1})
L-rhamnose monohydrate	10-500	y=(267989±8414)x-(1000000±1208906)	0.997	12.73	0.014	0.048
D-(+)-xylose	10-500	y=(296830±7543)x-(3000000±2000000)	0.998	6.19	0.009	0.031
D-(-)-fructose	10-500	y=(296830±88007)x-(3000000±2000000)	0.998	10.12	0.014	0.046
D-(+)-galactose	10-500	y = (464377±8946)x - (8000000±2000000)	0.998	1.86	0.002	0.008
D-(+)-glucose	10-500	y = (536016±9780)x - (9000000±1527525)	0.998	2.03	0.002	0.008
D-galacturonic acid monohydrate	10-500	y = (14120±836)x + (14094±29916)	0.999	13.02	0.393	1.309
D-glucuronic acid	10-500	y = (59686±5028)x - (2000000±577350)	0.990	3.13	0.059	0.195
myo-inositol	10-500	y = (830946±13513)x - (20000000±5773503)	0.998	1.51	0.001	0.005

R^2 – coefficient of determination; *CV* – coefficient of variation; *LOD* – limit of the determination; *LOQ* – limit of the quantification.

Table S4. Qualitative and quantitative (mean percentage content) carbohydrate composition of exopolysaccharide of three *R. leguminosarum* bv. *trifolii* strains isolated from root nodules of *T. repens* growing on 100-yrs old Zn-Pb waste heap Bolesław (4.1H, 5.2H, 7.2H) and four non-polluted reference grassland Bolestraszyce ones (3.9K, 4.4K, 5.10K, 8.8K) not exposed (control) and exposed to 0.05 mM and 1.0 mM Cd, 0.5 mM and 2.5 mM Zn and 0.5 mM and 2.5 mM Pb.

	4.4K				3.9K				8.8K				5.10K				7.2H				4.1H				5.2H			
**Carbohydrate**	**C**	**Cd 1.0**	**Zn 2.5**	**Pb 2.5**	**C**	**Cd 1.0**	**Zn 2.5**	**Pb 2.5**	**C**	**Cd 1.0**	**Zn 2.5**	**Pb 2.5**	**C**	**Cd 1.0**	**Zn 2.5**	**Pb 2.5**	**C**	**Cd 1.0**	**Zn 2.5**	**Pb 2.5**	**C**	**Cd 1.0**	**Zn 2.5**	**Pb 2.5**	**C**	**Cd 1.0**	**Zn 2.5**	**Pb 2.5**
α-D,L-lyxopyranose	-	-	-	-	-	-	-	-	-	-	-	-	-	-	-	-	-	-	-	-	0.25	-	-	-	-	-	-	-
α-D-arabinopyranose	-	-	-	-	-	-	-	-	-	-	-	-	-	0.04	-	-	-	-	-	-	-	-	-	-	-	-	-	-
D-rhamnose	-	-	-	-	-	-	-	-	-	-	-	-	-	-	-	-	-	-	-	-	-	-	-	-	-	-	-	-
L-rhamnose monohydrate	-	-	-	-	-	-	-	-	-	-	-	-	-	-	-	-	-	-	-	-	-	-	-	-	-	-	-	-
levoglucosan	1.34±0.21	-	-	-	0.99	-	-	-	-	-	-	-	-	-	-	-	-	-	-	-	-	-	-	-	-	-	-	-
6-deoxy-D-glucose	-	-	-	-	4.82	-	-	-	3.12±0.18a	-	-	-	-	-	-	-	-	-	-	-	3.13±0.74a	-	-	-	2.19	-	-	-
methyl α-D-mannopyranoside	-	1.05±0.37	1.02	1.95±0.21	0.30	1.55±0.07	-	1.87±0.15	0.17	1.64±0.46	0.19	1.77±0.40a	-	1.20±0.90a	-	2.06±0.20a	-	1.12±0.09	0.10	1.70±1.17	0.18±0.01	1.18±0.05	-	2.82±1.33	-	1.19±0.06	0.07	2.07a
galactofuranoside	1.28	-	-	-	0.50	-	-	-	0.31	-	-	-	-	-	-	-	-	-	-	-	0.32±0.06	-	-	-	0.26	-	-	-
α-D-mannopyranose	1.28±0.12	0.05±0a	-	0.10±0a	-	0.06±0a	-	0.10±0a	-	0.7	-	0.11	-	0.09	-	0.10 0	-	0.07±0a	-	0.09±0.03	-	0.05±0.01a	-	0.13±0.06	-	0.09±0.02a	-	-
D-fructose	-	0.22±0.06b	0.16	0.15±0.04b	1.44	0.08±0.04a	-	0.10±0a	1.07±0.02b	0.16	-	0.08	-	0.10	-	0.10±0.01	-	-	0.11	0.08±0.04	1.40±0.11b	-	0.11 0	0.15±0.13	0.95	-	0.09	-
β-D-galactopyranoside	1.15	-	-	-	-	-	-	-	0.17	-	-	-	-	0.24	-	-	-	-	-	-	0.18±0.06	-	-	-	0.21	-	-	-
α-D-galactofuranose	-	-	-	-	-	-	-	-	-	-	-	-	-	-	-	-	-	-	-	-	-	-	-	-	-	-	-	-
α-D-mannofuranose	-	-	-	-	-	-	-	-	-	-	-	-	-	-	-	-	-	-	-	-	-	-	-	-	-	-	-	-
α-glucofuranose	0.47[a]	-	-	-	0.65	-	-	-	0.36	-	-	-	-	-	-	-	-	-	-	-	0.45	-	-	-	0.49	-	-	-
D-galactose	-	-	-	-	-	-	-	-	15.09±0.24c	-	-	-	-	-	-	-	-	-	-	-	14.79±0.90c	-	-	-	11.71a	-	-	-
α-D-glucopyranoside	6.53±0.99[b]	0.18±0.01b	0.26	0.28±0.01c	3.61	0.17±0.03b	-	0.29±0.04b	0.38±0.24	0.23±0.02a	0.38	0.24±0.03b	3.99	0.28±0.23	-	0.26±0.01b	5.04±0.16	0.39±0.27	-	0.20±0.07	0.32±0.15	0.32±0.25a	-	0.31±0.11	4.70±0.49b	0.29±0.11a	-	0.59
α-D-glucopyranose	-	-	-	-	4.33	-	-	-	4.63±0.01a	-	-	-	-	-	-	-	-	-	-	-	4.72±0.23a	-	-	-	3.75b	-	-	-
D-glucose	0.77	-	-	-	4.71	-	-	-	5.17±0.31a	-	-	-	-	-	-	-	-	-	-	-	4.19±0.09a	-	-	-	3.35b	-	-	-
β-D-galactopyranose	-	0.06±0a	-	0.07a	0.25	0.07±0.01a	-	0.08±0.01a	0.28±0.04	0.08±0.01	-	0.08	-	0.15	-	0.08±0.01a	-	0.12±0.06a	-	0.06±0	0.22	0.11±0.02a	-	0.09±0.02	-	0.16±0.04a	-	0.17
β-D-mannopyranose	-	-	-	-	-	-	-	-	-	-	-	-	-	-	-	-	-	-	-	-	-	-	-	-	-	-	-	-
β-talopyranose	-	-	-	-	-	-	-	-	-	-	-	-	-	-	-	-	-	0.34	-	-	-	0.36a	-	-	-	0.29	-	0.10
sedoheptulose	-	1.60±0.90	-	-	0.47	4.04±2.93c*	-	-	0.53±0.04	2.26±1.36*	-	-	-	7.02±5.57b	-	0.74a	-	8.23±0.93b	-	-	0.58±0.04	8.49±1.02b*	-	-	0.59	9.19±0.19b*	0.46a	-
glucitol	79.78±3.49[c]	96.48±0.39c*	98.41±0.98a*	97.05±0.19d*	84.96±5.93a	93.60±2.97d*	99.41*	97.16±0.13c*	67.70±1.27d	95.34±2.06b*	99.26±0.05a*	97.48±0.67c*	95.49±6.39a	90.68±4.07c	99.52	96.64±0.28c	89.68±1.99a	89.58±1.37c	99.52±0.08a*	97.53±1.34*a	67.46±2.34d	92.18±1.18c*	99.55±0.03a*	96.21±1.61*a	77.61±11c	93.18±2.32c*	99.32±0.21b*	96.59b*
β-D-glucopyranose	7.28±0.78[b]	-	-	-	3.37±4.06	-	-	-	0.65±0.40	-	-	-	5.04	-	-	-	5.29±1.82	-	-	-	0.50±0.20	-	-	-	4.32±3.12	-	-	-
galactonic acid	-	-	-	-	-	-	-	-	-	-	-	-	-	-	-	-	-	-	-	-	0.12	-	-	-	0.65	-	-	-
gluconic acid	1.05±0.25	-	-	-	-	-	-	-	-	-	-	-	-	-	-	-	-	-	-	-	-	-	-	-	-	-	-	-
glucuronic acid	-	-	-	-	-	-	-	-	-	-	-	-	-	-	-	-	-	-	-	-	-	-	-	-	-	-	-	-
D-glucuronic acid	-	-	-	-	-	-	-	-	-	-	-	-	-	-	-	-	-	-	-	-	-	-	-	-	-	-	-	-

myo-inositol	-	-	-	-	-	-	-	-	-	-	-	-	-	-	-	-	-	-	-	-	-	-	-	-	-	-	-	-
N-acetyl-D-glucosamine	-	-	-	-	-	-	-	-	-	-	-	-	-	-	-	-	-	-	-	-	-	-	-	-	-	-	-	-

Results are presented as means ± SD for n= 2, analyzed with one-way analysis of variance (ANOVA), and significant differences between means were estimated with the usage of the multiple range Duncan's test. Letters are used to mark significance of differences intra particular rhizobia groups, asterisk is used to mark significant differences between rhizobia exposed to metals and non-exposed ones (C-group). A not detected compound was marked as a hyphen. A range of colours from green to red is proportional to the concentration increase of particular carbohydrates.

Table S5. Mean percentage of living and dead cells in biofilms formed by *R. leguminosarum* bv. *trifolii* isolated from root nodules of *T. repens* growing on 100-yrs old Zn-Pb waste heap Bolesław (H) and non-polluted reference grassland Bolestraszyce (K), not exposed (control) and exposed to 0.05 mM and 1.0 mM Cd, 0.5 mM and 2.5 mM Zn and 0.5 mM and 2.5 mM Pb.

**Rhizobia strain**	**control**		**Cd 0.05**		**Cd 1.0**		**Zn 0.5**		**Zn 2.5**		**Pb 0.5**		**Pb 2.5**	
	**L**	**D**	**L**	**D**	**L**	**D**	**L**	**D**	**L**	**D**	**L**	**D**	**L**	**D**
8.8K	76.16±1.70^{a}	23.84±1.70^{a}	66.72±2.16^{a}	33.28±2.16^{a}	27.27±4.80^{a}	72.73±4.80^{a}	24.01±1.55^{a}	75.99±1.55^{a}	34.29±4.72^{a}	65.71±4.72^{a}	68.60±3.46^{a}	31.40±3.46^{a}	32.31±5.14^{a}	67.69±5.14^{a}
4.4K	74.60±2.06^{a}	25.40±2.06^{a}	72.05±2.32^{a}	27.95±2.32^{a}	48.88±1.16^{b}	51.12±1.16^{b}	43.58±2.05^{b}	56.42±2.05^{b}	57.62±1.66^{b}	42.38±1.66^{b}	78.94±1.48^{b}	21.06±1.48^{b}	44.35±1.46^{b}	55.65±1.46^{b}
3.9K	71.57±0.49^{a}	28.43±0.49^{a}	59.69±1.60^{b}	41.31±1.60^{b}	18.50±0.62^{c}	81.50±0.62^{c}	27.71±2.97^{a}	72.29±2.97^{a}	22.15±5.03^{a}	77.85±5.03^{a}	61.98±3.33^{a}	38.02±3.53^{a}	26.56±3.49^{a}	73.44±3.49^{a}
5.10K	76.46±1.85^{a}	23.54±1.85^{a}	69.82±2.16^{a}	31.28±2.16^{a}	27.27±4.80^{a}	72.73±4.80^{a}	24.01±1.55^{a}	75.99±1.55^{a}	34.29±4.72^{a}	65.71±4.72^{a}	68.60±3.46^{a}	31.40±3.46^{a}	32.31±5.14^{a}	67.69±5.14^{a}
**$\bar{x}_K$**	**75.65±2.74**	**22.35±2.74**	**67.50±1.67#**	**32.50±1.67#**	**25.83±5.79##**	**75.17±5.79##**	**26.39±3.68#**	**73.61±3.68#**	**26.67±5.97#**	**73.33±5.97#**	**72.26±8.79#**	**27.24±8.79#**	**29.97±2.69##**	**71.03±2.69##**
5.2H	72.36±6.02$_{a}$	24.64±6.02$_{a}$	82.44±3.19$_{a}$	17.56±3.19$_{a}$	61.24±2.47$_{a}$	38.76±2.47$_{a}$	80.84±2.08$_{a}$	19.16±2.08$_{a}$	34.05±2.54$_{a}$	65.95±2.54$_{a}$	82.77±2.58$_{a}$	17.23±2.58$_{a}$	36.28±1.33$_{a}$	64.72±1.33$_{a}$
7.2H	75.04±3.63$_{a}$	24.96±3.63$_{a}$	73.10±4.11$_{b}$	26.90±4.11$_{b}$	54.47±3.49$_{b}$	45.53±3.49$_{b}$	72.49±3.09$_{b}$	27.51±3.09$_{b}$	57.83±3.85$_{b}$	43.17±3.35$_{b}$	78.36±3.15$_{a}$	21.64±3.15$_{a}$	42.28±1.58$_{b}$	57.72±1.58$_{b}$
4.1H	76.15±3.03$_{a}$	24.85±3.03$_{a}$	68.28±0.67$_{b}$	31.72±0.67$_{b}$	30.39±1.24$_{c}$	70.61±1.24$_{c}$	28.77±3.81$_{c}$	71.23±3.81$_{c}$	29.05±1.16$_{c}$	70.95±1.16$_{c}$	82.25$_{a}$	14.75$_{a}$	33.63±0.21$_{c}$	67.37±0.21$_{c}$
**$\bar{x}_H$**	**75.00±3.68**	**25.00±3.68**	**75.86±5.72***	**24.14±5.72***	**54.86±5.80*##**	**45.14±5.80*##**	**75.63±9.47***	**24.37±9.47***	**46.50±4.50*#**	**53.50±4.50*#**	**80.02±3.00**	**19.98±3.00**	**36.63±3.14*#**	**63.37±3.14*#**

Results are presented as means ± SD for n= (2-8), analyzed with one-way analysis of variance (ANOVA), and significant differences between means were estimated with the usage of the multiple range Duncan's. Abbreviations: $\bar{x}_K$ – mean living and dead cells of rhizobial non-polluted reference origin group, $\bar{x}_H$ – mean living and dead cells of rhizobial waste heap origin group. Asterisk is used to mark significance of differences between non-polluted reference and waste heap origin strains, letters in upper case are used to mark significance of differences among rhizobia of non-polluted reference origin, letters in lower case are used to indicate significance of differences among waste heap area origin ones, number sign is used to indicate the significance of differences between control and two doses of particular metal *i.e.*, Cd 0.05 *vs.* Cd 1.0, Zn 0.5 *vs.* Zn 2.5, and Pb 0.5 *vs.* Pb 2.5 groups. Density of colours proportional with an increase of dye-labelled cells was used to mark differences in living cell amounts (green) and dead ones (red) of non-polluted reference origin *R. leguminosarum* bv. *trifolii* strains (K) and waste heap ones (H).
